# What role do biocontrol agents with Mg^2+^ play in the fate of antibiotic resistome and pathogenic bacteria in the phyllosphere?

**DOI:** 10.1128/msystems.01126-23

**Published:** 2024-03-20

**Authors:** Qiqi Zhi, Ge Tan, Shaolong Wu, Qianqian Ma, Jianqiang Fan, Yiqiang Chen, Jingjing Li, Zhengrong Hu, Yansong Xiao, Liangzhi Li, Zhenghua Liu, Zhaoyue Yang, Zhendong Yang, Delong Meng, Huaqun Yin, Qianjun Tang, Tianbo Liu

**Affiliations:** 1 School of Minerals Processing and Bioengineering, Central South University, Changsha, China; 2 Key Laboratory of Biometallurgy, Ministry of Education, Changsha, China; 3 China Tobacco Hunan Industrial Co., Ltd., Changsha, China; 4 Tobacco Research Institute of Hunan Province, Changsha, China; 5 College of Plant Protection, Hunan Agricultural University, Changsha, China; 6 Technology Center, China Tobacco Fujian Industrial Co., Ltd, Xiamen, Fujian, China; 7 Chenzhou Tobacco Company of Hunan Province, Chenzhou, China; 8 School of Architecture and Civil Engineering, Chengdu University, Chengdu, Sichuan, China; University of California, Irvine, California, USA

**Keywords:** antibiotic resistance genes, biocontrol agents, mutation, evolution, homologous recombination, metagenome

## Abstract

The contamination of the plant phyllosphere with antibiotics and antibiotic resistance genes (ARGs), caused by application of antibiotics, is a significant environmental issue in agricultural management. Alternatively, biocontrol agents are environmentally friendly and have attracted a lot of interest. However, the influence of biocontrol agents on the phyllosphere resistome remains unknown. In this study, we applied biocontrol agents to control the wildfire disease in the Solanaceae crops and investigated their effects on the resistome and the pathogen in the phyllosphere by using metagenomics. A total of 250 ARGs were detected from 15 samples, which showed a variation in distribution across treatments of biocontrol agents (BA), BA with Mg^2+^ (T1), BA with Mn^2+^ (T2), and kasugamycin (T3) and nontreated (CK). The results showed that the abundance of ARGs under the treatment of BA-Mg^2+^ was lower than that in the CK group. The abundance of *cphA3* (carbapenem resistance), *PME-1* (carbapenem resistance), *tcr3* (tetracycline antibiotic resistance), and *AAC (3)-VIIIa* (aminoglycoside antibiotic resistance) in BA-Mg^2+^ was significantly higher than that in BA-Mn^2+^ (*P* < 0.05). The abundance of *cphA3*, *PME_1,* and *tcr3* was significantly negatively related to the abundance of the phyllosphere pathogen *Pseudomonas syringae* (*P* < 0.05). We also found that the upstream and downstream regions of *cphA3* were relatively conserved, in which *rpl*, *rpm,* and *rps* gene families were identified in most sequences (92%). The Ka/Ks of *cphA3* was 0 in all observed sequences, indicating that under the action of purifying selection, nonsynonymous substitutions are often gradually eliminated in the population. Overall, this study clarifies the effect of biocontrol agents with Mg^2+^ on the distribution of the phyllosphere resistome and provides evolutionary insights into the biocontrol process.

**IMPORTANCE:**

Our study applied metagenomics analysis to examine the impact of biocontrol agents (BAs) on the phyllosphere resistome and the pathogen. Irregular use of antibiotics has led to the escalating dissemination of antibiotic resistance genes (ARGs) in the environment. The majority of BA research has focused on the effect of monospecies on the plant disease control process, the role of the compound BA with nutrition elements in the phyllosphere disease, and the resistome is still unknown. We believe BAs are eco-friendly alternatives for antibiotics to combat the transfer of ARGs. Our results revealed that BA-Mg^2+^ had a lower relative abundance of ARGs compared to the CK group, and the phyllosphere pathogen *Pseudomonas syringae* was negatively related to three specific ARGs, *cphA3*, *PME-1,* and *tcr3*. These three genes also present different Ka/Ks. We believe that the identification of the distribution and evolution modes of ARGs further elucidates the ecological role and facilitates the development of BAs, which will attract general interest in this field.

## INTRODUCTION

Over the past few decades, the prevalence of antibiotic resistance mainly driven by the improper use of antibiotics has emerged as a global concern (1, 2) . Antibiotic use in agriculture to increase crop productivity can prompt the emergence of antibiotic resistance in the pathogens, which may reduce the effectiveness of antimicrobial therapy for agriculture production (1). Agricultural soil is a natural reservoir that stores antibiotic resistance genes (ARGs) and antibiotic-resistant bacteria (ARB) from both native microbes and those introduced by anthropogenic activities (3, 4). Besides, resistant bacteria can enter the plant root and migrate into the leaf and stem tissue, accelerating the spread of resistance genes in plants (1). ARG abundance may affect the plant community succession process, and the phyllosphere ARG profile is linked to the leaf bacteria and nutrient content (5). In addition, the abuse of antibiotics increases environmental pollution and builds up chemical residues in the treated ecosystem (6). Thereby, environmentally friendly biocontrol agents have been identified as hotspots for the inhibition of the dissemination of ARGs and ARB (7). Biocontrol agents (BAs) using microorganisms are a possible way to minimize pollution and greatly reduce the negative impacts of antibiotics on the environment (8). However, the mechanisms and key influencing factors in this control process remain unknown.

BAs, which contain living organisms, have been extensively studied for their capacity to promote plant growth (9). Furthermore, many of these BAs can also halt pathogen proliferation (10). Studies have revealed that biocontrol agents are under intensive study worldwide and are generally considered promising substitutes for chemical antibiotics in the crop disease control process (11). BA can interact with the plant, the targeted pathogen, and the resident microflora to improve their resistance to pathogen attack (12). BA could act directly through antagonistic effects on the pathogen, for example, antibiosis, parasitism, and reducing pathogen virulence through competition (13). In addition, biocontrol agents can induce or augment resistance against pathogen infections in plant tissues without directly interacting antagonistically with the pathogen (14). A wide variety of root-associated mutualists, including *Pseudomonas*, *Bacillus*, *Trichoderma*, and mycorrhiza species sensitize the plant immune system for enhanced defense without directly activating costly defenses (9). The foliar bacterial biocontrol agents and plant growth promoting rhizobacteria (PGPR) strains may induce plant resistance under field conditions, providing effective suppression of bacterial speck and spot of tomato (15). A potentially sustainable and eco-friendly way to control pathogens is the use of beneficial microorganisms that can promote plant growth and health (16, 17). These organisms may enhance the level and consistency of control by providing multiple mechanisms of action, a more stable rhizosphere community, and effective control over a wider range of environmental conditions (18). This control process should be systematic and should consider the dynamic succession of microbial communities. The probability that a beneficial bacterium will dominate the associated microbiota is higher when several bacteria are administered than when only one probiotic strain is involved (19) (19, 20). Actually, the resident microbial community is a resilient complex environment that could be sometimes hostile to the establishment of beneficial microorganisms (21). This situation can be avoided by the use of supporting beneficial additions that could promote diverse metabolic activities (BA establishment or development, strong ecological network, etc.) for improved biocontrol. Magnesium (Mg) and manganese (Mn), as essential mineral elements for plants and microbes, can have both indirect as well as direct effects on disease and nutrition in the phyllosphere (22, 23). Even though the research on microorganisms as potential biocontrol agents has increased, how compound biocontrol agents impact the resistome in the phyllosphere is still unknown, and now the direct evidence that BAs could combat the challenge of the occurrence and dissemination of resistance genes is lacking. *Pseudomonas syringae* is one of the most common pathogens that infect the Solanaceae phyllosphere (24, 25). *P. syringae* could cause canker disease, the infection symptoms of which include late winter die-back of young canes, frequently accompanied by rust red exudates from canes and trunks, and the presence of necrotic lesions with chlorotic halos on leaves during the spring (26). Kasugamycin (KSG) has been used as a major antibiotic to control various bacterial diseases, such as wildfire diseases in different crops (27–29). However, antibiotic-resistant bacterial strains have emerged because of the indiscriminate and long-term use of this antibiotic (30, 31). Promising achievements in terms of biological control against pathogens causing foliar diseases have emerged, especially after the successful use of certain antagonistic strains of *Pseudomonas* (32). A recent study showed that plant wildfire disease infection rates were significantly decreased by the foliar application of a bacterial mix of *Bacillus* (87.74%), *Alcaligenes* (7.69%), *Pseudochrobactrum* (2.86%), and *Achromobacter* (1.05%) (31). According to the former research, the combination of multiple compatible biocontrol organisms has proven to be effective in many cases; an example is the biocontrol of *Fusarium* by a combination of nonpathogenic strains of *F. oxysporum* and fluorescent strains of *Pseudomonas* (33). The microorganisms from the plant itself may be an ideal origin of BA to control plant disease, microorganisms isolated from the rhizospheric zone of an specific crop may be better adapted to that plant, and may provide better biological control than organisms isolated from other plants since they are already adapted to the plant or plant part as well as to the particular environmental conditions in which they must function (33). Although the development of adaptive functional biocontrol agents may be an alternative and eco-friendly method of controlling the disease, few studies have evaluated the distribution of ARGs in the phyllosphere.

Furthermore, antibiotic resistance is a classical evolutionary process based on a specific reaction (natural selection) by microbes to survive antibiotic exposure. However, ARGs occur in an extremely complex and variable ecobiological system encompassing the whole planet, involving numerous other causes (34). Antibiotic selective pressure can also increase the volume of ARGs in the environment and accelerate resistome evolution (35, 36). The probability of ARG evolution increases rapidly in “hot spots” of resistomes (37), it may signal instability and potential evolution of unexpected genes associations (20). However, the impact of biocontrol agents on the evolution of ARGs still remains unknown.

Thus, to better address these concerns and questions, a metagenomics analysis was performed. The aim of this study was to provide a deep and comprehensive evaluation of the correlation of ARGs and the phyllosphere pathogen *P. syringae* and the distribution and evolution of ARGs under treatments with biocontrol agents added with nutrition elements Mg/Mn, which could improve our understanding of the phyllosphere resistome and infer innovative approaches for controlling diseases on crop plants.

## MATERIALS AND METHODS

### Strain preparation

The antagonistic flora was isolated from the model Solanaceae crop tobacco leaf. The activated strain was fermented in a 50-L fermenter with 5% inoculum volume, pH 8, 180 r/min, and fermented at 35°C for 48 hours, and 10 L of fresh fermentation broth was pumped out into a sample bottle. The compound bacterial liquid was formulated into a compound microbial bacterial agent, and the bacterial concentration of the compound microbial bacterial agent was 10^8^–10^9^ cfu/mL. The concentration was dispensed into 500-mL bacteria collection bottles on an ultra-clean workbench with a liquid volume of 400 mL, then we refrigerated and centrifuged at 10,000 rpm for 10 min, and the bacteria was spread evenly into glass petri dishes. We sealed and placed in a −80°C refrigerator for 4–5 hours and then freeze-dried to obtain the bacterial powder. The powder was evenly added with additives such as 2% white carbon black carrier, 1% sodium lignosulfonate, 1% Tween 80, 0.2% K_2_HPO_4,_ and 0.01% carboxymethyl cellulose to obtain wettable biocontrol powder (BA). On the basis of wettable biocontrol agents (BA), 5% MgSO_4_ and 2% MnSO_4_ trace elements were added, respectively, to obtain multifunctional composite microbial biocontrol powders BA-Mg^2+^ and BA-Mn^2+^.

### Sample collection and experiment trail setup

In this study, the model Solanaceae crop tobacco leaf microbial samples were collected from Jiahe (lng: 112°22'12", lat: 25°34'48") in Chenzhou, Hunan Province. We set up four groups with three replicates in each group including CK, BA-Mg^2+^ (5% MgSO_4_, T1), BA-Mn^2+^ (2% MnSO_4_, T2), and 4% KSG (T3; Table 1). The negative control group CK applied water 5 L per plot; T1 and T2 were administered BA-Mg^2+^ and BA-Mn^2+^ powder to 5 L, respectively; the positive control group T3 was treated with 60 g KSG plus 2 L water, and the cell concentration after dilution was above 10^7^ cfu/mL. We checked the disease condition of the field and started using the medicine when sporadic lesions were first observed. The method of spraying was adopted to evenly spray on the front and back sides of the leaves. The spraying finished within 1 month. Seven days after the spraying was completed, leaf parts were collected in plastic bags. We drilled 15 g of leaf samples from different parts of the leaf surface using a sterile punch and added 50 mL of bacterial phosphate buffer (pH = 7.0). Then, the samples were shaken on a constant temperature shaker at 28°C at 170 rpm for 30 min, the process was repeated three times to collect the bacterial suspension, and centrifuged for 15 min (4°C, 10,000 rpm) to collect the microorganisms. The precipitate was suspended in sterile water and eluted three times. Finally, we resuspended the microorganisms in 1 mL of sterile water for subsequent DNA extraction. The leaves were rinsed with sterile water three times, and the last rinsing solution was applied to the LB plate medium and cultured at a constant temperature of 30°C for 2 days to judge whether the microorganisms on the surface of the leaves were completely eluted.

### DNA isolation, library construction, and sequencing

DNA from different samples was extracted using the E.Z.N.A. Stool DNA Kit (D4015-02, Omega, Inc., USA) according to the manufacturer’s instructions. The reagent which was designed to uncover DNA from trace amounts of the sample has been shown to be effective for the preparation of DNA of most bacteria. Sample blanks consisted of unused swabs processed through DNA extraction and tested to contain no DNA amplicons. The total DNA was eluted in 50 µl of elution buffer by a modification of the procedure described by the manufacturer (QIAGEN) and stored at −80°C. DNA library was constructed by using the TruSeq Nano DNA LT Library Preparation Kit (FC-121–4001). The DNA was fragmented by dsDNA Fragmentase (NEB, M0348S) by incubating at 37° C for 30 min. Library construction began with fragmented cDNA. Blunt-end DNA fragments were generated using a combination of fill-in reactions and exonuclease activity, and size selection was performed with the provided sample purification beads. An A-base was then added to the blunt ends of each strand, preparing them for ligation to the indexed adapters. Each adapter contained a T-base overhang for ligating the adapter to the A-tailed fragmented DNA. These adapters contained the full complement of sequencing primer hybridization sites for single, paired-end, and indexed reads. Single- or dual-index adapters were ligated to the fragments, and the ligated products were amplified with PCR by the following conditions: initial denaturation at 95°C for 3 min; eight cycles of denaturation at 98°C for 15 sec, annealing at 60°C for 15 sec, and extension at 72°C for 30 sec, and then final extension at 72°C for 5 min. At last, we performed the 2 × 150 bp paired-end sequencing (PE150) on an illumina Novaseq 6000 (LC-Bio Technology Co., Ltd., Hangzhou, China) following the vendor’s recommended protocol. All the sequencing data were deposited at the NCBI BioProject repository under accession number PRJNA983864.

### *De novo* metagenome assembly, gene annotation, and taxonomic analysis of metagenomic assemblies

The quality control of the raw data was performed using fastp software (38), including removing joints, repetitive sequences, and low-quality sequences, and the parameters were default parameters. These sequences were filtered by aligning the filtered data to the host genome (from NCBI) using Kneaddata v0.10.0 (http://huttenhower.sph.harvard.edu/kneaddata). Filtered reads from different treatments (438.32 GB) were assembled separately using metaWRAP v1.2.1 (39). The assembled contigs were organized into refined bins, and we selected 25 bins with high completeness (>70%) and low contamination (<5%) to construct the phylogenetic tree. The R package “ggplot2” v3.4.2 was applied to analyze the distribution of ARGs among different treatment groups (40). The multiple comparisons LSD test was applied to test the significance difference in ARG abundance in different treatments (41). The *t*-test significance difference was calculated by the R package “ggsignif” v0.6.4 (42). Taxonomic analysis of metagenomic assemblies using GTDB-TK v2.1.0 was applied (43). MMseqs2 v13.45111 was employed to cluster sequences to reduce sequence redundancy (44). Prokka v1.14.6 was used to annotate metagenomic reconstructions (45). ARGs were annotated using the CARD v1.2.0 and FARME database (46, 47).

### Identifying allele frequency differences, phylogenetic analysis, and co-occurrence network construction

The clean reads were mapped to the reference genomes using BBmap (https://sourceforge.net/projects/bbmap/), SNPs were identified using Varscan v2.3.9 with the thresholds of minor allele frequency being greater than 0.05, minimum read base quality of 20, strand-filter of 90%, and minimum read depth of 10 (48). Coverage was determined as the number of metagenomic reads mapping with 95% sequence identity to each gene. Gene coverage was normalized by gene length, gene frequency was determined for each gene by dividing its coverage by the median coverage of all genes within a genome bin, which implies the copy number of each gene per cell within a microbial population. The coverage was calculated by coverm v0.6.1 (https://github.com/wwood/CoverM). Phyloflash v3.4 was applied to analyze the phylogenetic pattern (49). The phylogenomic tree was produced with GToTree v1.6.12 (50). The R package “microeco” v 0.20.0 was used to construct the co-occurrence network and calculate the “Spearman” correlation among nodes (51). The R package “corrplot” v 0.92 was applied to analyze the “Pearson” correlation of ARGs and pathogens (52). The heatmap was analyzed by R package “pheatmap” v1.0.12 (53). The gene map was analyzed by R package gggenes v0.5.1 (https://cran.r-project.org/web/packages/gggenes). The Ka/Ks of sequences was calculated in https://services.cbu.uib.no/tools/kaks. The mOTUs v3.0.1 was applied to analyze the microbial composition of all treatment groups and biocontrol agents (54). The fitting was performed with “ggpmisc” v0.5.4 (55) and “ggpubr” v0.6.0 (56) in R.

## RESULTS

### Microbial community structure and the distribution of ARGs in the phyllosphere

Metagenomic analyses were conducted to investigate the changes in the foliar microbiome and resistome present after BA-Mg^2+^, BA-Mn^2+^, and KSG therapeutic treatments. As shown in Fig. S1, the microbial samples consisted of various strains of bacteria and were mainly composed of *Pseudomonas oryzihabitans*, *Pseudomonas coleopterorum/rhizosphaerae*, *Stenotrophomonas maltophilia*, *Sphingomonas aerolata*, and *Methylobacterium sp001424705*. The BA was mainly composed of *Pseudomonas oryzihabitans*, *Pseudomonas coleopterorum/rhizosphaerae*.

**Fig 1:**
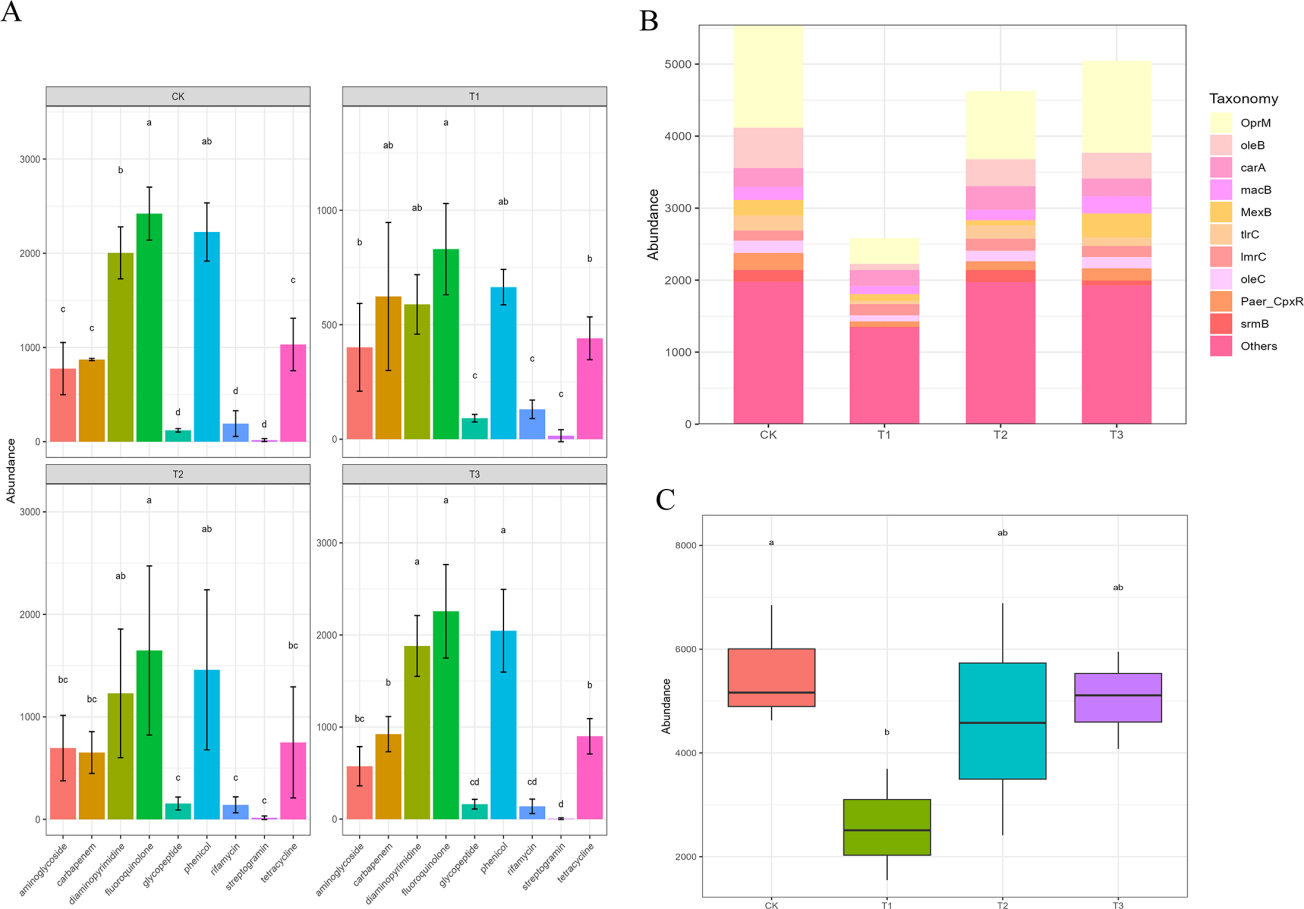
(**A**). Genes belonging to nine drug classes in different groups (LSD test; *P* < 0.05). (**B**). The composition and proportion of top 10 ARGs among four treatment groups. (**C**). The abundance of ARGs among four treatment groups.

The ARGs mostly resulted in resistance to nine drug classes, namely, aminoglycoside, tetracycline, fluoroquinolone, phenicol, diaminopyrimidine, glycopeptide, rifamycin, and carbapenem. Furthermore, the abundance of ARGs associated with fluoroquinolone resistance was higher than other drug class resistances (Fig. 1A). A total of 250 ARGs were detected from metagenomic reads across all samples. As shown in (Fig. 1B ; Table S1), the top 10 abundance of ARGs were involved in *srmB* (macrolide antibiotic), *Paer_CpxR* (multidrug resistance), *oleC* (macrolide antibiotic), *tlrC* (macrolide antibiotic; lincosamide antibiotic), *lmrC* (lincosamide antibiotic), *macB* (macrolide antibiotic), *MexB* (multidrug resistance), *carA* (macrolide antibiotic), *oleB* (macrolide antibiotic), and *OprM* (multidrug resistance). Further analyses showed that the abundance of ARGs in T1 was significantly lower than that in CK, implying that the BA-Mg^2+^ may have an effect on the distribution of ARGs (Fig. 1C; LSD test, *P* < 0.05); moreover, the abundance of *P. syringae* in T1 was lowest compared to other treatments (Fig. S3).

**Fig 2:**
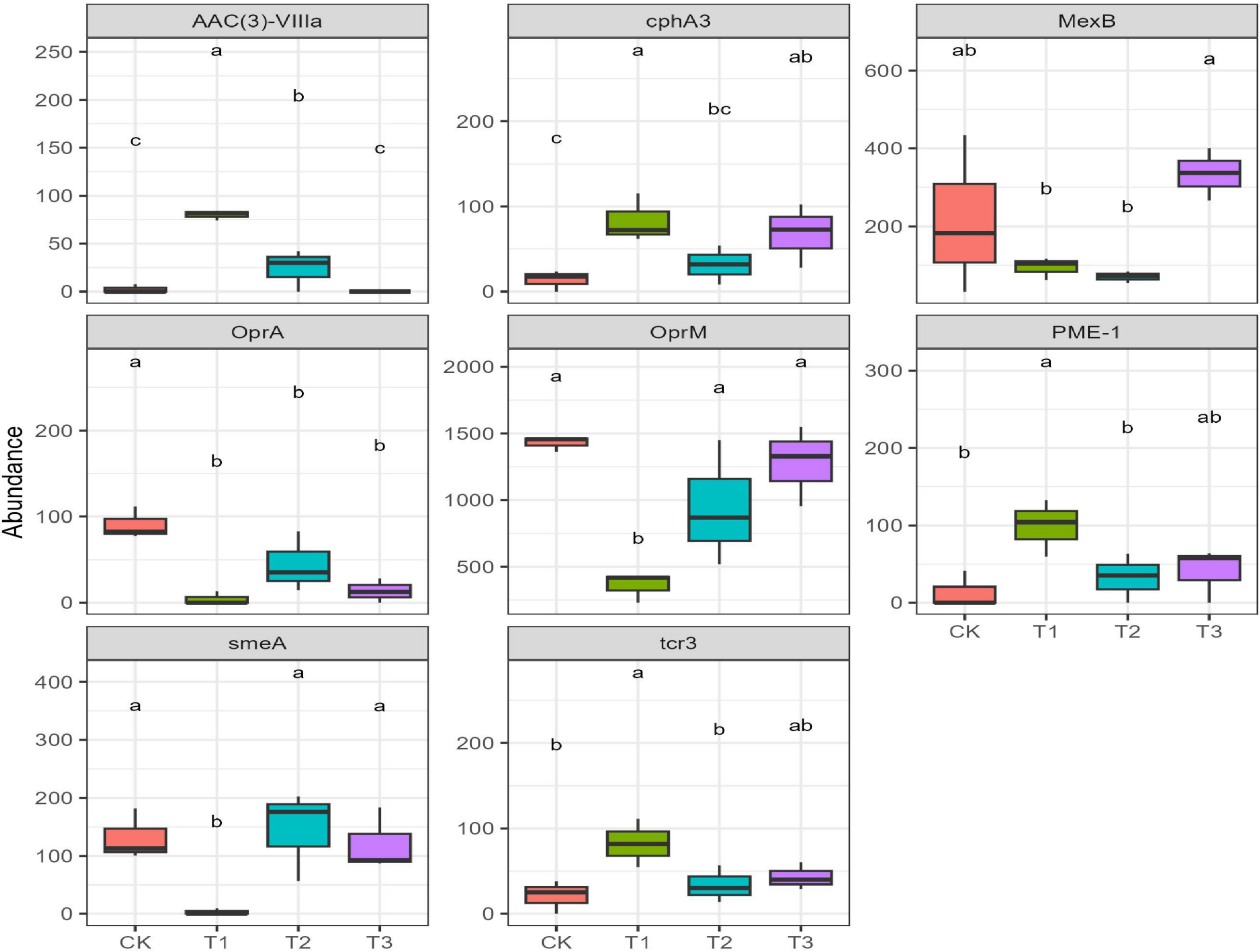
ARGs with a significant difference under different treatments (LSD test; *P* < 0.05).

The abundance of *MexB* (multidrug resistance) in T3 was significantly higher than that of T1 and T2 (Fig. 2; LSD test, *P* < 0.05). The abundance of *cphA3* (carbapenem resistance), *PME-1* (carbapenem resistance), *tcr3* (tetracycline antibiotic resistance), and *AAC (3)-VIIIa* (aminoglycoside antibiotic resistance) in T1 was significantly higher than that of CK and T2 (Fig. 2; LSD test, *P* < 0.05). The abundance of *smeA* (penam, aminoglycoside, and cephamycin resistance) and *OprM* (tetracycline and macrolide antibiotic resistance) in T1 was lower than that in other groups, while the abundance of *OprA* (multidrug resistance) in CK was higher than that in other groups (Fig. 2).

### *De novo* metagenomes assembly and phylogenetic analysis

A total of 46 metagenome-assembly genomes (MAGs) were recovered from metagenomic reads, among which five MAGs were recovered from metagenome in CK, 10 MAGs were recovered from metagenome in T1, seven MAGs were recovered from metagenome in T2, and 24 MAGs were recovered from metagenome in T3. The 25 MAGs have high completeness (>70%) and low contamination (<5%). The results of phylogenetic analyses showed that 25 bins belong to Proteobacteria, including *Sphingomonas* (10), *Pseudomonas* (9), *Stenotrophomonas* (2), *Kosakonia* (1), *Pantoea* (1), *Xanthomonas* (1), and *Methylobacterium* (1).

**Fig 3:**
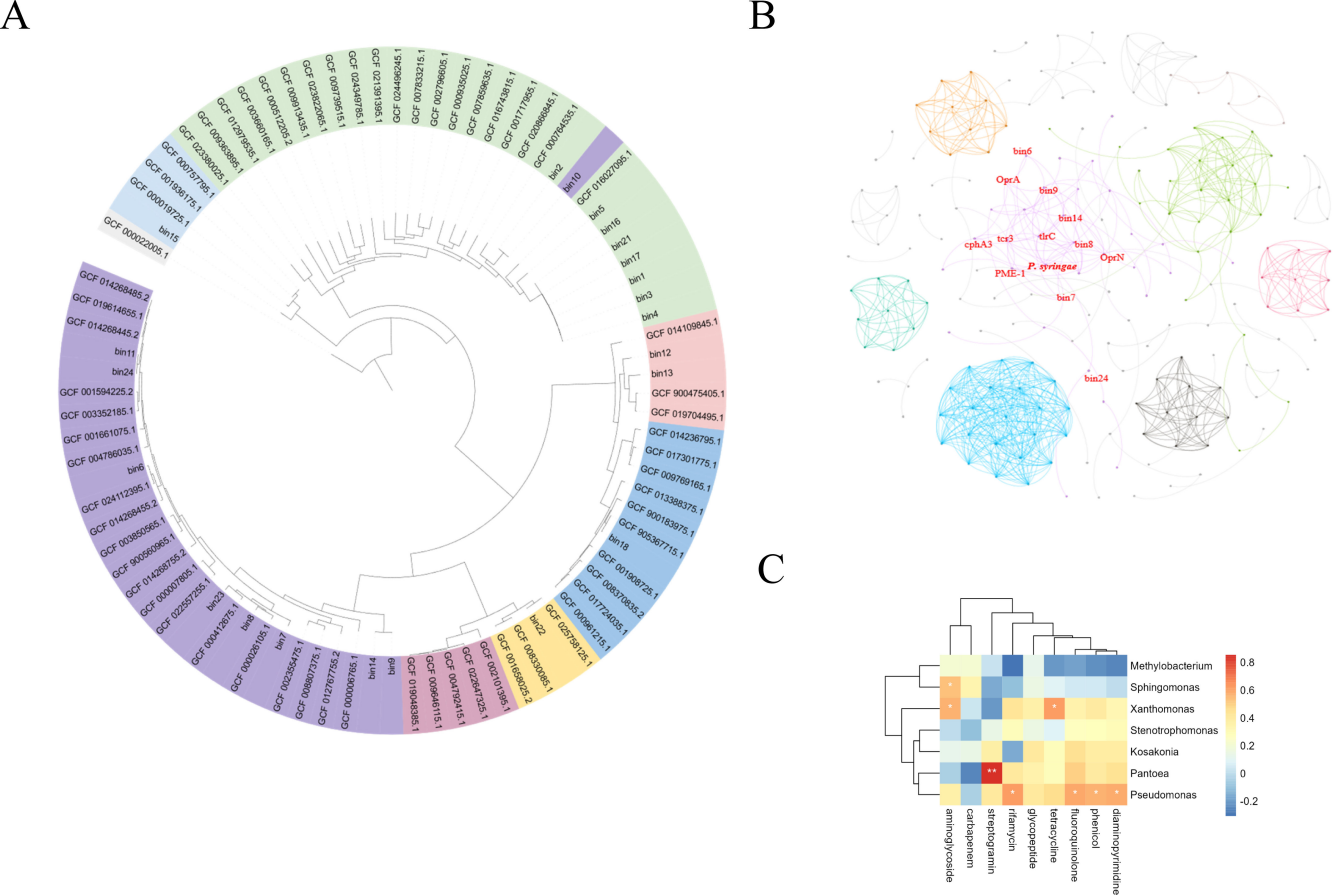
(**A**) The phylogenetic assignment of assembled genome bins; organisms were colored based on the genus level. (**B**) The co-occurrence network of antibiotic-resistant genes. (**C**) The heatmap of binnings and ARGs belonging to different drug classes.

The phylogenetic analysis suggested that these 25 bins all belong to Proteobacteria. As shown in Table S3 and the phylogenetic tree (Fig. 3A; Table S2), 21 of total bins were classified at the species level, and all were classified at the genus level; nine bins belonged to *Pseudomonas*, one bin belonged to *Kosakonia*, one bin belonged to *Pantoea*, one bin belonged to *Xanthomonas*, 10 bins belonged to *Sphingomonas*, one bin belonged to *Methylobacterium*, and two bins belonged to *Stenotrophomonas*.

### The co-occurrence network of antibiotic-resistant genes with *P. syringae* and the evolution of *cphA3*, *PME-1,* and *tcr3*

**Fig 4:**
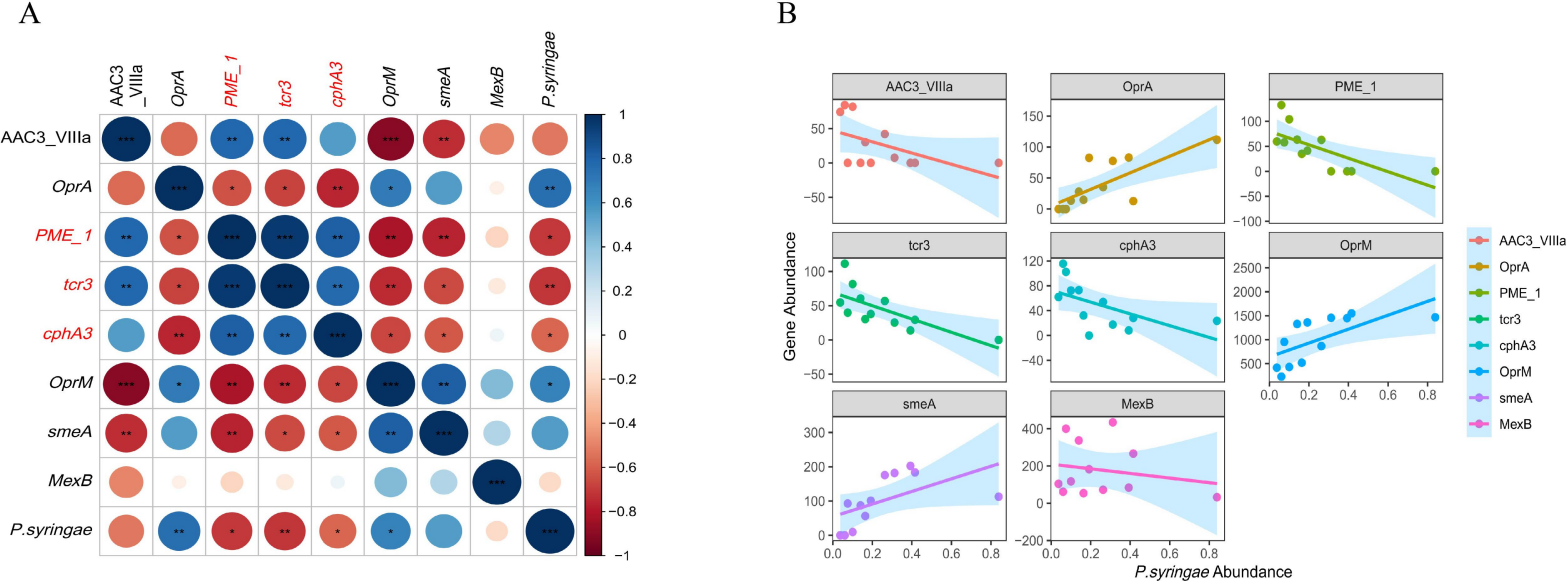
(**A**) The correlation between *P. syringae* and ARGs (Pearson correlation, *P* < 0.05). (**B**) The lm of the abundance of ARGs and *P. syringae*. The blue shadow indicates the 95% confidence interval.

Network analysis is often used to analyze the potential relationships between bacterial communities and target genes in an environment. According to the results of correlation analysis, the most important correlation between ARGs, MGEs, and *Pseudomonas syringae* was analyzed with *P* < 0.05 and correlation coefficient > 0.3 as the screening criteria. The *OprA*, *cphA3*, *PME-1*, *tcr3*, *tlrc*, and *OprN* were significantly related to *Pseudomonas simiae*, *Pseudomonas oryzihabitans*, *Pseudomonas sp002843585,* and *Pseudomonas syringae* in the same module (Fig. 3B and C) The abundance of *cphA3*, *PME_1,* and *tcr3* was significantly negatively related to the abundance of *Pseudomonas syringae* (*P* < 0.05; Fig. 4). Bin6 had the closest development distance to *P. syringae* among all binnings (Fig. S2).

**Fig 5:**
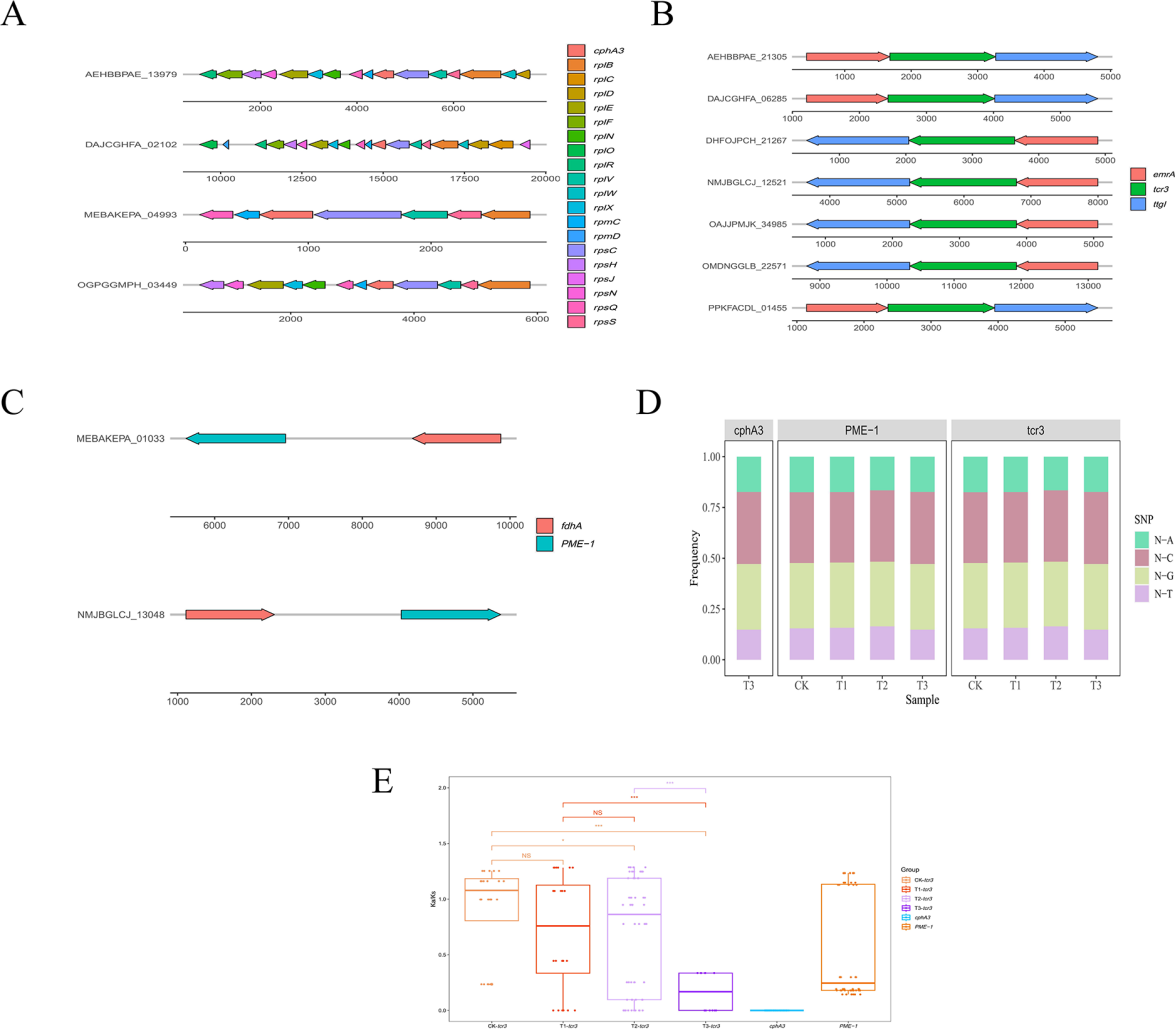
(**A to C**) The main structure type of *cphA3*, *tcr3,* and *PME-1* sequences in the metagenome. The arrows denote genes, and arrow length denotes gene size. (**D**) The proportion of SNPs N-A, N-T, N-C, and N-G in different sequences. E The Ka/Ks of *cphA3*, *tcr3,* and *PME-1* (*t* test; *P* < 0.05).

The upstream and downstream regions of *cphA3* were relatively conservative, in which the *rpl*, *rpm,* and *rps* gene families were identified in most sequences (92%). In seven of the total 90 sequences, the *emrA* and *ttgI* were located in the upstream and downstream regions of *tcr3*. Additionally, only two of the 36 total sequences contained *fdhA* alongside the *PME-1*, showing that *tcr3* and *PME-1* were less conservative than *cphA3*. (Fig. 5A through C). For *PME-1* and *tcr3*, the ratio of N-A, N-C, and N-G was similar in CK, T1, T2, and T3; for *cphA3*, the SNP only swept in T3 (antibiotic treatments) (Fig. 5D). The *tcr3* had the significantly lowest Ka/Ks in T3 compared to that of other treatments, while *cphA3* had a Ka/Ks of 0 in all different treatments (Fig. 5E; *t* test; *P* < 0.01).

### SNP identification and homologous recombination of the microbial community

Genes related to both transporter and recombination in cells were covered by SNPs (Table S3). To understand the effects of these variant alleles, genes covered by these SNPs were extracted for functional module annotation and pathway analysis, and the results suggested that these mutations were mainly related to transmembrane transport and DNA homologous recombination, such as *SSB_1*, *RecF*, *DpoIII,* and other SOS response factors (Fig. S4).

## DISCUSSION

### The biocontrol agents with Mg^2+^ contributed to the distribution of the phyllosphere resistome

In this study, metagenomic analyses were applied to reveal the effects of biocontrol agents on the distribution of the phyllosphere resistome. We found that the abundance of ARGs and *P. syringae* in the BA-Mg^2+^ treatment was lowest compared to other groups. In addition, the results showed that the abundance of *cphA3*, *PME-1,* and *tcr3* in BA-Mg^2+^ remained significantly higher than that in other groups. Remarkably, the abundance of *cphA3* , *PME_1,* and *tcr3* was significantly negatively related to the abundance of *P. syringae,* indicating that the BA-Mg^2+^ may exert an antibiotic-like effect on the pathogens. A number of examples in which ARG is associated with lower virulence, for example, *Acinetobacter* strains, and porin-deficient carbapenem-resistant or multidrug-resistant *P. aeruginosa* (with resistance in the last involving efflux pumps) are less virulent in the clinical setting (57, 58). The BA, mainly composed of *Pseudomonas* spp., which acted as functional microorganisms, limits the migration and colonization of the pathogen and directly inhibits the horizontal and vertical transfer of its ARGs; moreover, the resident response of the ARG-poor microbiome reduces the space of microorganisms with a high ARG microbiome, thus indirectly limiting the abundance of ARGs in indigenous communities (59). Studies have shown that Mg^2+^ could facilitate the synthesis of carbapenem, which has a destructive effect on the cell wall of pathogens; furthermore, it could also impact the synthesis of the glutamine synthetase in *P. syringae* (60) (61). Adequate supply of Mg nutrients promoted the accumulation of amino acids and total sugars in plants (62). The abundance of *smeA* and *OprM* related to multidrug resistance in BA-Mg^2+^ was lower than that in BA-Mn^2+^, suggesting that the BA-Mg^2+^ and BA-Mn^2+^ may exert different effects to control pathogens. Moreover, BA-Mg^2+^ may activate more specific pathways like the expression of β-lactamase among foliar microbial communities, and be more effective in controlling the pathogen than BA-Mn^2+^, which is worthy of further study.

### The evolution of the *cphA3* and *PME-1* related to beta-lactamase

The evolution of most ARGs is the evolution of particular changes in gene sequences, resulting in amino acid changes that increase or expand the host organism’s fitness to the environment (63). As shown in this study, the evolution of upstream and downstream regions of *cphA3* was more conservative than that of *PME-1* and *cphA3,* which was affected by the action of purifying selection, under which nonsynonymous substitutions are often gradually eliminated in the population. Most of these enzymes are probably derived from actinomycete ancestors, and horizontal transfer after gene capture in integrons, transposons, and conjugates has possibly contributed to allelic diversification (58). The acquisition of transposons, integrons, and gene cassettes by competent disparate species (64) and the possibility of acquiring large fragments and antimicrobial-resistant genes frequently occur (65). Horizontal gene transfer (HGT) provides the theoretical possibility for each gene of the biosphere to make contact with the genome of any bacterial organism; moreover, the role of HGT in evolution of the phyllosphere resistome needs deeper study.

### Mutation of the antibiotic efflux pump and homologous recombination results in the uptake and integration of ARGs

Gene-dosing and higher transcription effects increase the efficiency of chromosomally encoded efflux pumps; high-level expression can be achieved through mutation in their regulatory elements (63). Previous studies have demonstrated that overexpression of AdeABC, AdeFGH, or AdeIJK, which are three major resistance–nodulation–cell division (RND) pumps, contributes to antibiotic resistance in clinical strains of *Acinetobacter baumannii* (66–68). In the case of antibiotic resistance, identification of SNPs promotes the tracing of resistance conferring genes and the evolutionary counterpart of the bacterial resistome (69, 70). As revealed in this study, multiple membrane transporters, such as outer membrane efflux protein *BepC*, putative efflux pump periplasmic linker *TtgA*, vitamin B12 transporter *BtuB*, outer-membrane lipoprotein carrier protein *LolA*, and outer membrane protein *OprM,* are covered by SNPs and indels. The Ka/Ks of *tcr3*, a tetracycline efflux pump, was lower in T3 than in other groups, indicating that under the treatment of kasugamycin, nonsynonymous substitutions are often gradually eliminated in the population. The antibiotic therapy may promote selective pressure on specific ARGs, especially efflux pumps (71).

Recombination is a crucial approach employed by the microbiome to uptake and integrate exogenous DNA fragments into the host genome (72). Mutation essentially depends on the error rate of replication set by the accuracy of DNA polymerases and various DNA repair systems (63). This study has revealed that some homologous recombination genes were covered by SNP indels, such as *ssb_1*, *cbpA*, *dsbB*, *dnaQ*, *recF_1*, IS*Bcen27*, *grpE*, *secA*, *dnaB_1*, IS*Rel10*, *fhuA*, and SOS response factor IS*NYC*, suggesting that variations in recombination systems may facilitate the influx of ARGs as well as the accumulation of beneficial mutations in genotypically cohesive populations, which is consistent with previous studies (73, 74). While selective pressure in general can cause global transcriptional responses in bacteria and activate general stress responses such as the SOS response, many other genes are also frequently differentially expressed (72, 75). Studies have found that all antibiotics targeting DNA replication in bacteria (*S. pneumoniae*, *Escherichia coli*, *Bacillus cereus*, and *Staphylococcus aureus*) cause stalled replication forks, while DNA replication initiation continues. This results in an increase in copy numbers of genes close to the origin of replication and subsequent global changes in transcription. This shifted gene-dosage results in activation of the competence pathway, which thereby allows the bacterium to take up foreign DNA and potentially acquire antibiotic-resistance genes (72). Moreover, studies have revealed that recombination events are responsible for the mosaic structure of multiple ARGs, such as genes encoding penicillin-binding proteins (PBPs) in *S. pneumoniae* (73), ribosomal protection proteins (RBPs) in *Megasphaera elsdenii*, and *aph (3′)-IIa* in *P. aeruginosa* (76). Several epidemiological studies have reported that recombination mediates the distribution of transposons and integrative conjugative elements (ICEs) that carry antibiotic-resistant determinants (77, 78). In *Streptococci* and *Enterococci*, IS*1216*-mediated recombination plays an important role in the dissemination of *optrA* (77, 79) . There are potential regulatory effects between recombination and ARGs, facilitating the transfer and mutation of exogenous genes that confer antibiotic resistance, which will be further revealed by future studies.

### Conclusion

In summary, we scrutinized the significant impact of biocontrol agents on the resistome and pathogen in the phyllosphere through metagenome analysis. The abundance of *cphA3*, *PME_1,* and *tcr3* was significantly negatively related to the abundance of *P. syringae* (*P* < 0.05). The upstream and downstream regions of *cphA3* were relatively conservative, in which *rpl*, *rpm,* and *rps* gene families were identified in most sequences (92%). The Ka/Ks of *cphA3* was 0 in all observed sequences, indicating that under the action of purifying selection, nonsynonymous substitutions are often gradually eliminated in the population. Furthermore, the SNP analysis showed that the genes related to transporter and recombination genes were covered by SNPs in cells.

The findings from this study will directly contribute to a deeper understanding and strategic use of functional biocontrol agents. However, the mechanisms by which ARGs disseminate across the plant microbiome and affect the phyllosphere pathogen should be further explored in additional field studies. Future research could focus on the ecological effect of biocontrol agents on community assemblage and the evolution of the resistome.

**TABLE 1:** The treatments on the phyllosphere

Group	Treatment
CK	Water; three repeats
T1	BA-Mg^2+^; three repeats
T2	BA-Mn^2+^; three repeats
T3	kasugamycin (KSG); three repeats

## Data Availability

All the sequencing data were deposited at the NCBI BioProject repository under accession number PRJNA983864.

## SUPPLEMENTAL MATERIAL

Supplemental figures and tables msystems.01126-23-s0001.pdf Fig. S1 to S4 and Tables S1 to S3.

ASM does not own the copyrights to Supplemental Material that may be linked to, or accessed through, an article. The authors have granted ASM a non-exclusive, world-wide license to publish the Supplemental Material files. Please contact the corresponding author directly for reuse.
