# Supplementary material for: What role do biocontrol agents with Mg2+ play in the fate of antibiotic resistome and pathogenic bacteria in the phyllosphere?
Source: mSystems. 2024 Mar 20;9(4):e01126-23. doi: 10.1128/msystems.01126-23 (PMC11019836; doi:10.1128/msystems.01126-23)
Supplement: Supplemental figures and tables — Fig. S1 to S4 and Tables S1 to S3. [file msystems.01126-23-s0001.pdf]

Supplemental Figures and Tables

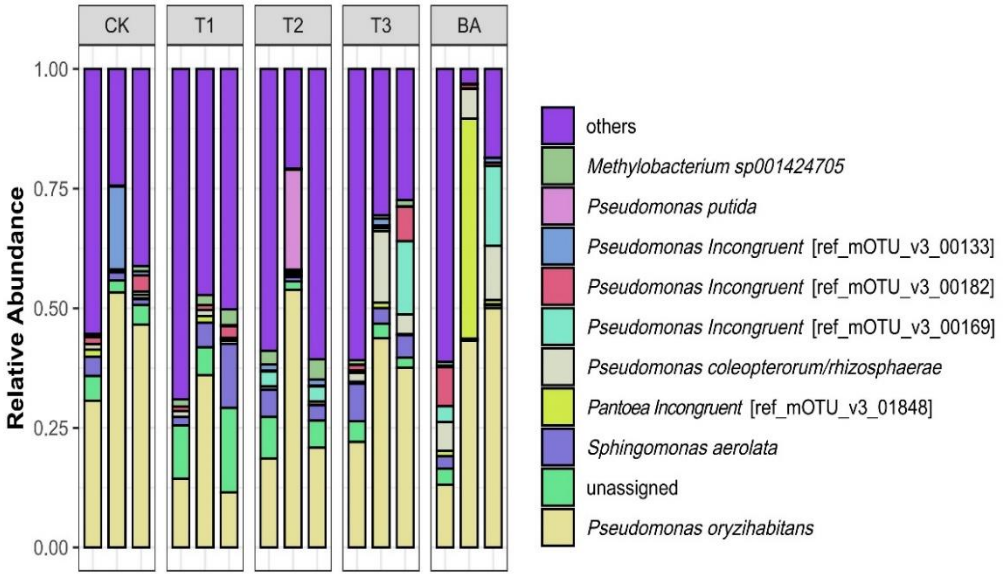

Fig. S1 The microbiome composition of different samples.

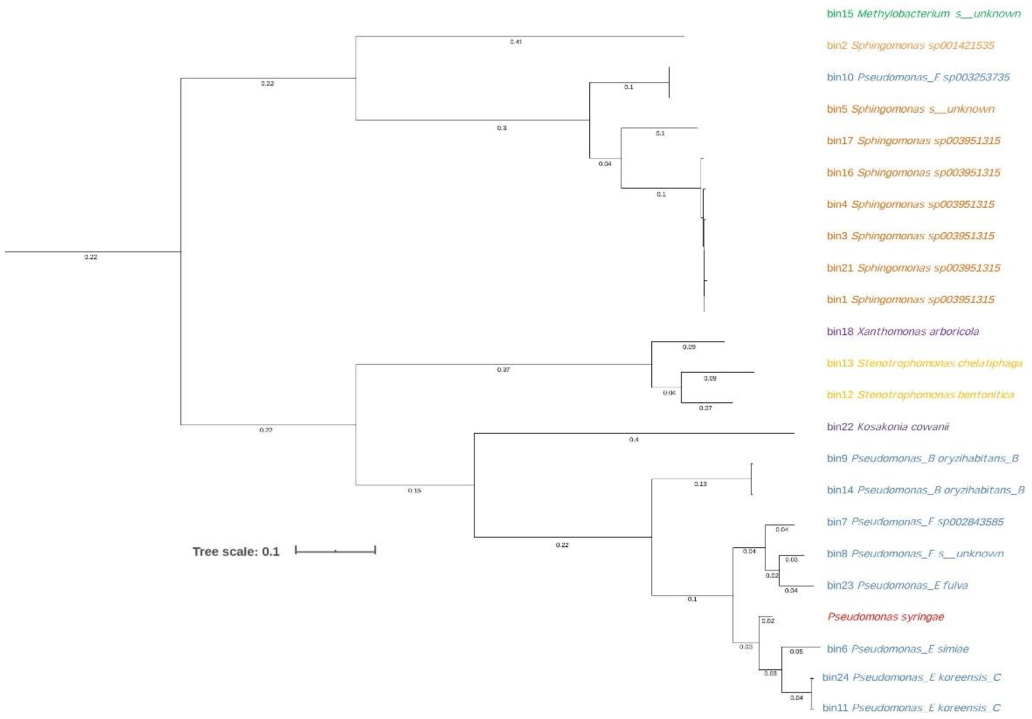

Fig. S2 The phylogenetic assignment of assembled genome bins and *P. syringae*.

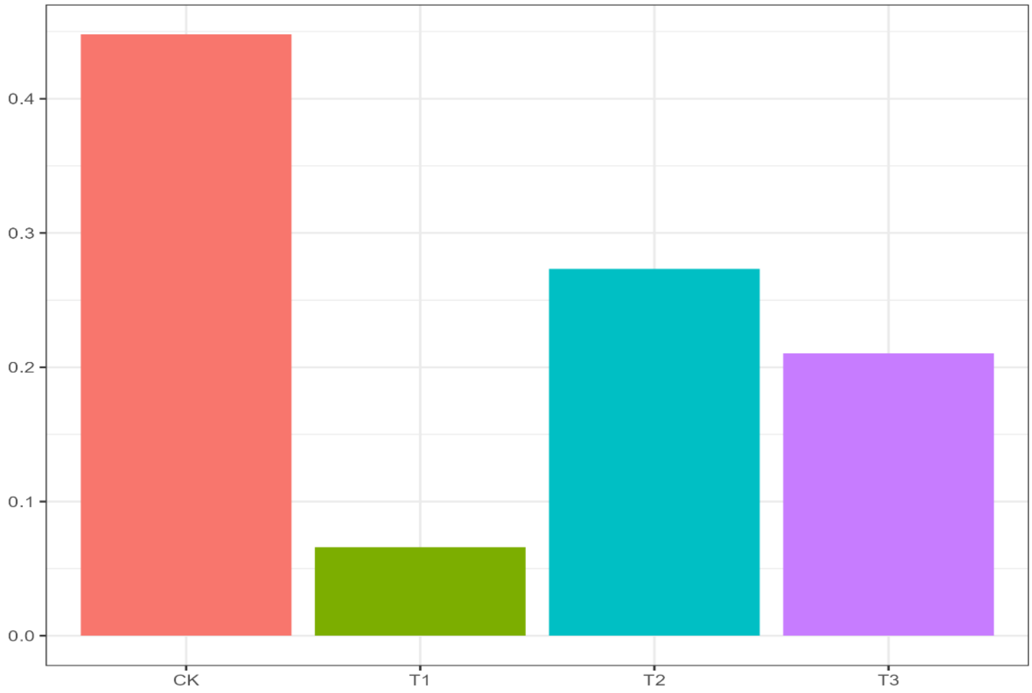

Fig. S3 The abundance of *P. syringae* in different treatments

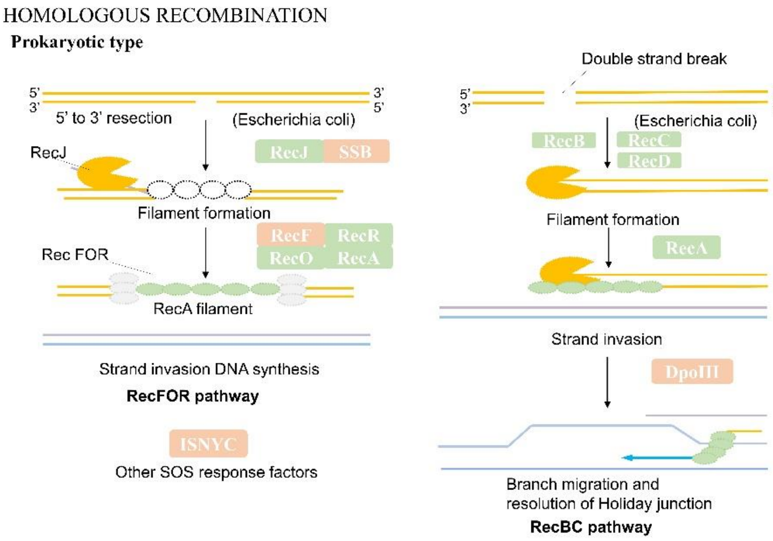

Fig. S4 Homologous Recombination pathways covered by significant SNPs. Genes covered by SNPs with constant allele frequency changes were denoted by reddish shading.

Table. S1 The top 10 abundance of ARGs in each sample

**ID**	**ARO**	**Gene**
X63451.1	ARO:3002828	*srmB*
LT673656.1	ARO:3004054	*Paer_CpxR*
L06249.1	ARO:3003748	*oleC*
M57437.1	ARO:3002827	*tlrC*
EU124663.1	ARO:3002881	*lmrC*
AY768532.1	ARO:3000535	*macB*
L11616.1	ARO:3000378	*MexB*
L36601.1	ARO:3003036	*oleB*
M80346.1	ARO:3002817	*carA*
AE004091.2	ARO:3000805	*OprN*
AE004091.2	ARO:3000377	*MexA*
AE004091.2	ARO:3000809	*OpmD*
AE004091.2	ARO:3000803	*MexE*
AE004091.2	ARO:3000149	*FosA*
AE004091.2	ARO:3000807	*MexH*
AE004091.2	ARO:3002985	*arnA*
AE004091.2	ARO:3003679	*TriA*
AE004091.2	ARO:3003680	*TriB*
AE004091.2	ARO:3003681	*TriC*
AE004091.2	ARO:3003682	*OpmH*
AE004091.2	ARO:3003692	*MexJ*
AE004091.2	ARO:3003693	*MexK*
AE004091.2	ARO:3003030	*MexV*
AE004091.2	ARO:3004073	*MuxA*
AE004091.2	ARO:3004072	*OpmB*
AE004091.2	ARO:3004038	*Paer_emrE*
AE004091.2	ARO:3004056	*ArmR*
AE004091.2	ARO:3000804	*MexF*
AE004091.2	ARO:3000379	*OprM*
AE004091.2	ARO:3004074	*MuxB*
AE004091.2	ARO:3004075	*MuxC*
AE004091.2	ARO:3004077	*PmpM*
AE004091.2	ARO:3004107	*Paer_soxR*
AE004091.2	ARO:3005063	*cprR*
AE004091.2	ARO:3005064	*cprS*
AE004091.2	ARO:3005067	*ParS*
AE004091.2	ARO:3005068	*ParR*
AE004091.2	ARO:3000806	*MexG*
AE004091.2	ARO:3000808	*MexI*
AE004091.2	ARO:3003710	*MexL*

Table. S2 The classification of 25 genome bins

Genome bin	Classification
1	d__Bacteria;p__Proteobacteria;c__Alphaproteobacteria;o__Sphingomonadales;f__Sphingomonadaceae;*g__Sphingomonas;s__Sphingomonas sp003951315*
2	d__Bacteria;p__Proteobacteria;c__Alphaproteobacteria;o__Sphingomonadales;f__Sphingomonadaceae;*g__Sphingomonas;s__Sphingomonas sp001421535*
3	d__Bacteria;p__Proteobacteria;c__Alphaproteobacteria;o__Sphingomonadales;f__Sphingomonadaceae;*g__Sphingomonas;s__Sphingomonas sp003951315*
4	d__Bacteria;p__Proteobacteria;c__Alphaproteobacteria;o__Sphingomonadales;f__Sphingomonadaceae;*g__Sphingomonas;s__Sphingomonas sp003951315*
5	d__Bacteria;p__Proteobacteria;c__Alphaproteobacteria;o__Sphingomonadales;f__Sphingomonadaceae;*g__Sphingomonas;s__*
6	d__Bacteria;p__Proteobacteria;c__Gammaproteobacteria;o__Pseudomonadales;f__Pseudomonadaceae;*g__Pseudomonas_E;s__Pseudomonas_E simiae*
7	d__Bacteria;p__Proteobacteria;c__Gammaproteobacteria;o__Pseudomonadales;f__Pseudomonadaceae;*g__Pseudomonas_E;s__Pseudomonas_E sp002843585*
8	d__Bacteria;p__Proteobacteria;c__Gammaproteobacteria;o__Pseudomonadales;f__Pseudomonadaceae;*g__Pseudomonas_E;s__*
9	d__Bacteria;p__Proteobacteria;c__Gammaproteobacteria;o__Pseudomonadales;f__Pseudomonadaceae;*g__Pseudomonas_B;s__Pseudomonas_B oryzihabitans_B*
10	d__Bacteria;p__Proteobacteria;c__Gammaproteobacteria;o__Pseudomonadales;f__Pseudomonadaceae;*g__Pseudomonas_E;s__Pseudomonas_E sp003253735*
11	d__Bacteria;p__Proteobacteria;c__Gammaproteobacteria;o__Pseudomonadales;f__Pseudomonadaceae;*g__Pseudomonas_E;s__Pseudomonas_E koreensis_C*
12	d__Bacteria;p__Proteobacteria;c__Gammaproteobacteria;o__Xanthomonadales;f__Xanthomonadaceae;*g__Stenotrophomonas;s__Stenotrophomonas bentonitica*
13	d__Bacteria;p__Proteobacteria;c__Gammaproteobacteria;o__Xanthomonadales;f__Xanthomonadaceae;*g__Stenotrophomonas;s__Stenotrophomonas chelatiphaga*
14	d__Bacteria;p__Proteobacteria;c__Gammaproteobacteria;o__Pseudomonadales;f__Pseudomonadaceae;*g__Pseudomonas_B;s__Pseudomonas_B oryzihabitans_B*
15	d__Bacteria;p__Proteobacteria;c__Alphaproteobacteria;o__Rhizobiales;f__Beijerinckiaceae;*g__Methylobacterium;s__*
16	d__Bacteria;p__Proteobacteria;c__Alphaproteobacteria;o__Sphingomonadales;f__Sphingomonadaceae;*g__Sphingomonas;s__Sphingomonas sp003951315*
17	d__Bacteria;p__Proteobacteria;c__Alphaproteobacteria;o__Sphingomonadales;f__Sphingomonadaceae;*g__Sphingomonas;s__Sphingomonas sp003951315*
18	d__Bacteria;p__Proteobacteria;c__Gammaproteobacteria;o__Xanthomonadales;f__Xanthomonadaceae;*g__Xanthomonas;s__Xanthomonas arboricola*
19	d__Bacteria;p__Proteobacteria;c__Gammaproteobacteria;o__Enterobacterales;f__Enterobacteriaceae;*g__Pantoea;s__Pantoea endophytica*
20	d__Bacteria;p__Proteobacteria;c__Alphaproteobacteria;o__Sphingomonadales;f__Sphingomonadaceae;*g__Sphingomonas;s__Sphingomonas sp003951315*
21	d__Bacteria;p__Proteobacteria;c__Alphaproteobacteria;o__Sphingomonadales;f__Sphingomonadaceae;*g__Sphingomonas;s__Sphingomonas sp003951315*

22	d__Bacteria;p__Proteobacteria;c__Gammaproteobacteria;o__Enterobacterales;f__Enterobacteriaceae;*g__Kosakonia;s__Kosakonia cowanii*
23	d__Bacteria;p__Proteobacteria;c__Gammaproteobacteria;o__Pseudomonadales;f__Pseudomonadaceae;*g__Pseudomonas_E;s__Pseudomonas_E fulva*
24	d__Bacteria;p__Proteobacteria;c__Gammaproteobacteria;o__Pseudomonadales;f__Pseudomonadaceae;*g__Pseudomonas_E;s__Pseudomonas_E koreensis_C*
25	d__Bacteria;p__Proteobacteria;c__Alphaproteobacteria;o__Sphingomonadales;f__Sphingomonadaceae;*g__Sphingomonas;s__*

Table. S3 The genes related with ARGs covered by SNPs

Gene	
**Transporter**	
lolA	Outer-membrane lipoprotein carrier protein
bepC_2	Outer membrane efflux protein BepC
ttgA	putative efflux pump periplasmic linker TtgA
lolA	Outer-membrane lipoprotein carrier protein
btuB_16	Vitamin B12 transporter BtuB
lolA	Outer-membrane lipoprotein carrier protein
oprM_2	Outer membrane protein OprM
bepC	Outer membrane efflux protein BepC
ISRel10	ISNCY family transposase ISRel10
btuB_10	Vitamin B12 transporter BtuB
bepC_3	Outer membrane efflux protein BepC
oprM_2	Outer membrane protein OprM
**Recombination**	
ssb_1	Single-stranded DNA-binding protein
cbpA	DNA-binding protein
dsbB	Disulfide bond formation protein B
dnaQ	DNA polymerase III is a complex multi-chain fermenter responsible for most of the breakdown synthesis in bacteria
recF_1	DNA replication and repair protein RecF
ISBcen27	ISNCY family transposase ISBcen27
grpE	Single-stranded DNA-binding protein
ssb	Single-stranded DNA-binding protein
secA	Protein translocase subunit SecA
dnaB_1	Replicative DNA helicase

ISRel10	ISNCY family transposase ISRel10
fhuA	Ferrichrome outer membrane transporter/phage receptor
**ARG**	
mdtC	Multidrug resistance protein MdtC
